# The effect of family sex composition on fertility desires and family planning behaviors in urban Uttar Pradesh, India

**DOI:** 10.1186/1742-4755-10-48

**Published:** 2013-09-11

**Authors:** Lisa M Calhoun, Priya Nanda, Ilene S Speizer, Meenakshi Jain

**Affiliations:** 1 Carolina Population Center, The University of North Carolina at Chapel Hill, Chapel Hill, NC, USA; 2 International Center for Research on Women (ICRW), Asia Regional Office, New Delhi, India; 3 Department of Maternal and Child Health, Gillings School of Global Public Health, The University of North Carolina at Chapel Hill, Chapel Hill, NC, USA; 4 FHI-360, Urban Health Initiative, New Delhi, India

**Keywords:** Family sex composition, Fertility desires, Family planning use, Uttar Pradesh, Urban

## Abstract

**Background:**

A cultural preference for sons has been well documented in India, resulting in skewed sex ratios, especially exhibited in northwest India. Previous research has shown that family sex composition is associated with family planning (FP) use and couples’ desire for more children. This study examines family sex composition and fertility and FP behaviors in urban Uttar Pradesh, India; little work has examined these issues in urban settings where family sizes are smaller and FP use is common.

**Methods:**

Data for this analysis comes from a 2010 representative survey of married, non-pregnant fecund women aged 15–49 from six cities in Uttar Pradesh, India. Multivariate analyses are used to examine the association between family sex composition and fertility desires and FP use.

**Results:**

The multivariate results indicate that family sex composition is associated with fertility desires and FP use. Women without living children and without at least one child of each sex are significantly less likely to want no more children and women with both sons and daughters but more sons are significantly more likely to want no more children as compared to women that have both sons and daughters but more daughters. Women with no living children and women with daughters but no sons are less likely to be modern FP users than nonusers whereas women with both sons and daughters but more sons are more likely to be modern FP users than nonusers as compared to women with both sons and daughters but more daughters.

**Conclusions:**

These findings confirm that family sex composition affects fertility behavior and also reveals that preference for sons persists in urban Uttar Pradesh. These results underscore the importance of programs and policies that work to enhance the value of girl children.

## Background

Recent research suggests that Asia has an estimated deficit of 60 million women, measured by the projected number of women compared with the actual number of women [1,2]. While girls have a biological advantage in survival over boys from conception onwards, in some Asian countries this biological advantage is reversed by a strong preference for sons, resulting in higher mortality of girls before birth or during early childhood and therefore increasingly skewed sex ratios at birth (SRB) and childhood [2,3]. This excess mortality of girls has been referred to as a deficit of girls in the region or Asia’s “missing” women [4,5]. All countries of Asia do not contribute equally to this deficit of girls; China and India are the main contributors [2,5].

An underlying reason for the deficit of girls is a preference for sons, which refers to the attitude that sons are more important and valuable than daughters. Parents in India have strong economic and social incentives to prefer sons over daughters, including important religious roles that only sons can perform, land inheritance to sons, sons’ role in old age support of parents, and perpetuation of the family name. These norms create a gap between the value of daughters to their natal homes and the value of adult women to their marital homes. Daughters, once married off, are considered the ‘property’ of their marital homes, and will not benefit her natal home. An adult woman, on the other hand, is perceived to bring value to her marital home through her contribution of labour and care [6]. Thus, even when adult women are economically productive or considered valuable for other reasons, daughters are not necessarily considered valuable. Researchers argue that the patriarchal family systems in India (particularly northwest India) are inflexible and this may contribute to the persistence of son preference [7].

Parents use discriminatory actions to manipulate the number of sons and daughters they have so as to attain their ideal family composition. This discrimination —whether prenatal or postnatal— is often associated with the sex composition of surviving children. Prenatal sex determination has been banned in India since the passing of the pre-conception and pre-natal diagnostic techniques (PCPNDT) act in 1994 [8], though the practice of pre-natal sex determination is still common [2]. Studies in Asia have shown that prenatal sex determination is more common at later birth orders among multiparous couples with mostly girl children [9]. In terms of postnatal discrimination, childhood mortality rates are commonly higher for girls than for boys in South Asia [10], though childhood mortality has been shown to be higher among girls with older sisters than among those without [11-15]. This suggests that neglect of girl children is focused on girls in families that already have girl children [11,12]. Further, girls in families in India with a strong son preference generally have worse outcomes in terms of health seeking behaviors, immunizations, schooling, and nutrition [11,16-22], though studies show that both boys and girls born after multiple siblings of the same sex also experience poorer health outcomes [13,23]. These findings highlight the need to consider the family composition of living children when exploring women’s preference for male children.

A commonly used approach to studying son preference investigates the association between family sex composition and actual fertility behaviors, such as family planning (FP) use or fertility desires. Globally, research on the association between family sex composition and fertility behavior is not conclusive. A multi-country analysis using DHS data by Arnold (1992), including countries in Asia, Africa, and Latin America, showed little evidence that family sex composition was associated with FP use, though data from India was not included in the analysis [24]. Other studies from Bangladesh, Vietnam and Nepal have shown little to no relationship between sex composition and FP use [25-28]. Despite the lack of evidence globally for an association between family sex composition and fertility behavior, there are a number of studies within India that show a significant relationship. A subsequent analysis focused on India by Arnold *et al.* (1998) showed that family composition affected fertility behaviors; in every state studied, including UP, among families with two children, those with two sons were more likely to use FP and less likely to want more children than those with two daughters [22]. Another recent study using Demographic and Health Survey data from multiple South Asian countries, including India, showed that women with both more children and more sons were more likely to use FP; within India, this relationship was stronger in northern states than in South India or West Bengal [29]. A similar analysis was carried out using the Indian National Family Health Survey (NFHS) data from 1992–93, and also showed that the family sex composition is associated with FP use, particularly among women that have two or more living sons [30]. A study from Madhya Pradesh showed that women with two daughters were more likely to want another child than other family compositions that included any number of sons [31]. Cross-sectional surveys and evaluations between 1985 and 1990 in rural South India showed that in areas with high FP use, couples preferred to have two sons, with or without a daughter before using FP, but in areas with lower FP use, couples had two sons and a daughter before using FP [32].

To date, much of the research on the association between son preference and reproductive outcomes in India has focused on rural settings; there is a void of research investigating this topic in urban Indian settings. Important distinctions exist between rural and urban areas which could influence son preference. Urban areas offer women greater opportunities for employment, education, and additionally more flexibility with respect to cultural norms, which may reduce the preference for sons [7]. Urban areas are often thought to offer an advantage through greater availability of healthcare, and particularly medical technology from the substantial public and private sectors [33]. This allows easier availability to sex selective technology in cities. In Uttar Pradesh (UP), India, the setting of this study, urban women are more likely to be users of modern FP, deliver in a health facility, fully immunize their children, and participate in household decision-making than rural women from this state [34]. Despite the “urban advantage” for many health indicators, the child sex ratio (number of girls per 1000 boys aged 0–6 years) is far more skewed in urban areas of India than in rural areas; in urban Uttar Pradesh, the child sex ratio is 879 girls per 1000 boys, which is much more adverse than it is in rural areas, at 904 girls per 1000 boys [35]. Clearly son preference remains an important issue in urban Uttar Pradesh where contraceptive prevalence is on the rise and total fertility rates are on the decline. There is a need to better understand the role of son preference on fertility desires and family planning use in urban Uttar Pradesh, which is the largest state in India and facing continued rapid urbanization [35].

This study uses recently collected data to fill an important gap in the understanding of the consequences of son preference in urban Uttar Pradesh, India. The objective of the study is to examine the association between family sex composition and fertility desires and FP use among currently married women.

## Methods

This study uses baseline data from the Measurement, Learning & Evaluation Project (MLE) for the Urban Reproductive Health Initiative (URHI) being implemented in eleven cities of UP. The URHI project in UP, the Urban Health Initiative (UHI), is being led by FHI360; this project was initially implemented in four cities, and expanded to seven more after the start of program implementation. The MLE project was tasked with rigorous impact evaluation of the URHI programs; the design includes a longitudinal survey of a large, representative sample of women in six cities of UP (Agra, Aligarh, Allahabad, Gorakhpur, Moradabad and Varanasi) as well as service delivery point surveys and cross-sectional survey data for men. The study cities were selected by the UHI program in collaboration with the Government of UP and the Government of India based on a number of criteria, including prioritization under government schemes, geographic and regional diversity, inclusion of big cities (>900,000) and medium cities (~750,000) based on population size, large slum populations, and low contraceptive prevalence. These criteria were prioritized as they are all critical in order to facilitate sustainability as well as replication and scale-up to other cities in UP. Based on provisional urban totals from district-level data in UP, the urban population in these six districts have a combined estimated population of approximately 8.7 million people, which is nearly 20% of the approximately 44.5 million urban residents state-wide [35].

Baseline data were collected in 2010 in Agra, Aligarh, Allahabad, Gorakhpur, Moradabad, and Varanasi [36], with the objective of selecting a representative sample of about 3,000 women per city. In order to construct a sampling frame, this study used lists of registered slums, satellite imagery and “ground-truthing” to identify slum settlements and develop maps of slum locations in each city. The spatial imagery was used to further identify slum settlements based on the density of the housing, the roofing type of the housing, and the road and path networks. Ground truthing was then undertaken to confirm the accuracy of the spatial imagery designation. The GIS data for the slum settlements was sent to the University of North Carolina, and the slum areas were then divided through mapping into primary sampling units (PSU) of approximately 100 households. The remaining areas of the city that were not identified as slums were then divided through mapping into PSUs of approximately 100 households for the non-slum sampling frame. This methodology therefore results in a sample of slum PSUs as well as non-slum PSUs which are more varied in terms of housing and neighborhood composition.

Based on the list of slum and non-slum PSUs, a representative sample was selected of 64 slum and 64 non-slum PSUs in each city; an equal number of slum and non-slum PSUs were selected to oversample slum areas for analyses that focus on the poorest women. Sample weights are used to adjust for the oversampling of slums in order make a representative sample at the city level. Using sample weights, the slum population accounts for a lower percentage of sampled women in Gorakhpur (9.7%), Allahabad (10.8%), and Moradabad (13.2%) and a higher percentage of sampled women in Agra (25.0%), Aligarh (18.7%), and Varanasi (26.1%).

In all selected PSUs, listing and mapping activities were carried out to number and identify all households. Based on the list of households in each PSU, a random sample of 30 households was selected. Selected households were approached for interview and the household head was consented for study participation. In selected households, all currently married women aged 15–49 who spent the previous night in the selected households were eligible for interview and approached for participation. Upon consenting to participate, women were asked questions about demographic characteristics, reproduction, FP use, fertility desires, maternal and child health and media exposure.

Female interviewers were employed to interview female respondents, as the subject matter of the questionnaire included sensitive information about reproductive health and women’s empowerment. Female interviewers had a graduate level education, were conversant in the local language, from the state of UP and had prior survey experience. Male supervisors oversaw a team of three female interviewers, and at times administered the household interview. Interviewers and supervisors underwent a 10-day training covering consent procedures, research ethics, questionnaire content, mock calls, and field practice. Additionally, supervisors underwent three extra days of training to review field check procedures and check-lists. All members of the field team signed a confidentiality agreement as a condition of employment on the project.

This project was approved by the Futures Group India Institutional Review Board and Institutional Review Boards at The University of North Carolina at Chapel Hill and the International Center for Research on Women. All study participants gave oral informed consent. A total of 17,643 currently married female respondents had complete interviews from baseline data collection in the six cities.

The key outcomes of interest are desire for more children and FP use. The analysis of desire for more children excludes women who were pregnant at the time of interview (n =1,208), were menopausal, were infecund or had had a hysterectomy (n = 1,510), and a small number of women with missing data for background characteristics (n = 47). From this subset of women, five women had missing information on desire for more children and were excluded. Analyses exploring desire for more children further excluded women who reported that they or their spouse had undergone sterilization (n = 3,875); the total analysis sample is 11,022 women for weighted analyses and 11,014 women for unweighted analyses. The outcome variable was based on a question on women’s desire for additional children and was coded as: does not want any more children versus wants more. Women who were undecided, responded that they did not have a preference, or said they did not know their spouse’s preference were coded as “wants more” because these women are unlikely to actively try to prevent a pregnancy (n = 21).

The second outcome variable of interest is women’s use of family planning. This analysis was limited to women who were non-pregnant at the time of interview, were not menopausal, were fecund, had not had a hysterectomy and women with no missing demographic information. This yielded 15,036 women for weighted analysis and 14,886 women for the unweighted analysis of FP use. Women were asked if they were currently using a method of FP, and among users, which method they were using. Family planning use was categorized into modern FP users, traditional FP users, and non-users. Modern FP use includes female and male sterilization, IUD, pills, condoms, injections, implants, and lactational amenorrhea (LAM). Traditional FP use includes rhythm, periodic abstinence and withdrawal.

The key independent variable of interest, family sex composition, is based on each individual’s number of living children and the sex of these children. Family sex composition was categorized into six categories: a) no living children; b) zero sons, one or more daughters; c) zero daughters, one or more sons; d) equal number of sons and daughters; e) have both sons and daughters, but have more daughters than sons; and f) have both sons and daughters, but have more sons than daughters. See Table 1 for the classification and distribution of this variable.

**Table 1:** Percentage distribution of married, non-pregnant fecund women aged 15–49, by selected characteristics

**Characteristic**	Desire for more children*	FP use*
**Age group**		
15–24	21.7	16.4
25–29	25.9	21.6
30–34	22.2	21.6
35–39	16.7	19.8
40+	13.5	20.6
**Education**		
None	27.9	31.2
1–11 years	33.1	34.8
12+ complete	39.1	34.0
**Employed in the past 12 months**		
Yes	12.2	12.9
No	87.8	87.1
**Wealth quintile (six city)**		
Poorest	21.2	21.1
Poor	20.7	20.4
Medium	20.1	20.6
Rich	19.3	19.4
Richest	18.7	18.6
**Caste**		
Scheduled caste/tribe	17.4	18.5
Other backward/extreme backward caste	42.9	42.6
None	39.7	38.9
**Religion**		
Muslim	23.9	20.5
non-Muslim	76.1	79.5
**City**		
Agra	24.2	23.9
Aligarh	13.1	11.4
Allahabad	19.0	19.4
Gorakhpur	15.1	15.6
Moradabad	9.7	9.2
Varanasi	18.9	20.6
**Number of live births**		
0–1	31.1	23.0
2	30.0	26.9
3	15.9	20.2
4+	23.0	30.0
**Family sex composition**		
No living children	10.6	7.8
Zero sons, one or more daughters	17.7	13.8
Zero daughters, one or more sons	23.2	21.5
Equal number of sons and daughters	21.7	21.9
Have both sons and daughters, but have more daughters than sons	14.2	17.1
Have both sons and daughters, but have more sons than daughters	12.6	17.9
**Total**	**100.0**	**100.0**
**Number of women (weighted)**	**11,022**	**15,036**

*All percentages and n’s are weighted.

A number of demographic variables were included in the multivariate analysis, including parity (0–1, 2, 3, 4+); education (no education, 1–11 years education, and 12+ years of education); age group (15–24, 25–29, 30–34, 35–29, 40+); religion (Muslim or non-Muslim); caste (scheduled caste or tribe, other backward caste or extremely backward caste, none); city (Agra, Aligarh, Allahabad, Gorakhpur, Moradabad, Varanasi); residence (slum; non-slum); and women’s employment status in the past one year (employed; not employed). Wealth was also included as an independent variable. The wealth index was created across the 6 cities based on 27 household assets and housing characteristics. The wealth quintiles were created using principal components analysis, as done by Filmer and Pritchett (2001) [37]. Women in the six cities were grouped into five categories: poorest, poor, medium, rich, and richest.

Univariate and bivariate analyses are weighted using the full-sample weights across the six cities and adjusted for clustering in the sample, using the *svy* commands in Stata. Bivariate analysis was used to explore the relationship between family sex composition and desire for children and FP use. Multivariate analyses were carried out unweighted as the goal of these analyses is to examine relationships between the variables; all multivariate analyses adjust for clustering in the sample. Multivariate logistic regression was used to determine if family sex composition was independently associated with women’s desire to have no more children, controlling for key demographic characteristics. Multinomial logistic regression models were used to explore the association between family sex composition and FP use, controlling for key demographic characteristics. All analyses were performed using Stata version 12.

## Results

Demographic characteristics of the currently married, non-pregnant women included in the analyses are presented in Table 1. Among women included in both samples, about a third had no education, a third had 1–11 years of education, and a third had 12+ years of education. Further, about 13% of women were currently employed and about one-fifth were Muslim and around 18% was a scheduled caste. Approximately 10 to 20% of women were from each city, with Moradabad accounting for around 9.2 to 9.7% of the sample. This may be due to Moradabad being a smaller city compared to the other cities, and therefore when weighted, it is a smaller sample of women as compared to the other five cities. Among the 11,022 women included in the desire for more children analysis, 10.6% had no living children, 17.7% had no sons, but had one or more daughters, 23.2% had no daughters, but had one or more sons, 21.7% had an equal number of sons and daughters, 14.2% had both sons and daughters, but had more daughters than sons and 12.6% had both sons and daughters but had more sons than daughters. Similarly, among the 15,036 women included in the FP use analysis, 7.8% had no living children, 13.8% had no sons, but had one or more daughters, 21.5% had no daughters, but had one or more sons, 21.9% had an equal number of sons and daughters, 17.1% had both sons and daughters, but had more daughters than sons and 17.9% had both sons and daughters but had more sons than daughters.

The bivariate results for desire for more children and FP use by family sex composition are shown in Table 2. Overall, 64.9% of women want no more children; differences are observed by family sex composition. In families with no living children, only 0.3% of women responded that they want no more children. Among the three categories of family sex composition that have both sons and daughters, greater than 90% of women want no more children in all three categories. Only one-third of women with zero sons and one or more daughters responded that they wanted no more children, whereas more than half of women with zero daughters and one or more sons responded that they want no more children. These differences were significant at p ≤ 0.001.

**Table 2:** Percentage of married, non-pregnant fecund women aged 15–49 and their desire for more children and percentage using family planning methods, by family sex composition

**Variables**	**No living children**	**Zero sons, one or more daughters**	**Zero daughters, one or more sons**	**Equal number of sons and daughters**	**Have both sons and daughters, but have more daughters than sons**	**Have both sons and daughters, but have more sons than daughters**	**Total**
**Desire for another child** (n = 11,022)*‡							
Want more children/undecided/do not know spouse’s desire	99.7	66.1	43.8	8.3	5.3	1.5	35.1
Want no more	0.3	33.9	56.2	91.8	94.8	98.5	64.9
**FP Use** (n = 15,036)*‡							
Modern	6.6	46.0	57.0	62.1	63.6	69.5	56.0
Traditional	3.5	21.7	19.5	19.6	16.9	15.5	17.4
Non-use	89.9	32.3	23.6	18.3	19.6	15.0	26.6
**Total**	100.0	100.0	100.0	100.0	100.0	100.0	100.0

*All percentages and n’s are weighted; ‡F statistic p-value is p ≤ .001.

Overall, modern FP use among women was 56.0%. Modern FP use was lowest among women with no living children at 6.6%. Forty-six percent of women with no living sons but one or more daughters were using modern FP, as compared to 57% of women with no living daughters but one or more sons. Modern FP use was similar among women with an equal number of sons and daughters and women that have both sons and daughters but have more daughters than sons, at 62.1% and 63.6% respectively. Modern FP use was the highest, at 69.5%, among women that have both sons and daughters but have more sons than daughters. With the exception of women with no living children, traditional method use was similar for all five remaining categories of family sex composition, ranging from 15.5–21.7%. The chi square p-value is p ≤ 0.001, indicating that FP use differs by family sex composition.

Multivariate logistic regression results exploring the patterns of desire for no more children by family sex composition, controlling for key demographic factors are presented in Table 3. Women with no living children, zero sons and one or more daughters, and zero daughters and one or more sons are all significantly less likely to want no more children compared to women that have both sons and daughters but more daughters than sons. Women that have both sons and daughters but more sons than daughters are significantly more likely to want no more children as compared to women that have both sons and daughters but more daughters than sons. The model in Table 3 controls for demographic factors which generally went in the expected directions. Notably, younger women, less educated women, lower parity women and Muslim women were significantly less likely to want no more children. Women that were employed in the past 12 months were significantly more likely to want no more children as compared to women that were not employed in the past 12 months. Interactions were explored between family sex composition and education and family sex composition and religion and were not found to be significant, and therefore are not included in the final model.

**Table 3:** Multivariate logistic regression findings for desire for more children among married, non-pregnant fecund women aged 15–49

**Variables**	**Want no more vs. want more children /undecided**
	**OR (95% CI)**
**Family sex composition**	
No living children	0.01 (0.00–0.02)***
Zero sons, one or more daughters	0.10 (0.07–0.13)***
Zero daughters, one or more sons	0.44 (0.33–0.58)***
Equal number of sons and daughters	0.98 (0.72–1.34)
Have both sons and daughters, but have more daughters than sons (ref)	1.00
Have both sons and daughters, but have more sons than daughters	3.73 (2.40–5.81)***
**Age group**	
15–24	0.04 (0.03–0.06)***
25–29	0.07 (0.05–0.11)***
30–34	0.13 (0.09–0.21)***
35–39	0.33 (0.21–0.53)***
40+ (ref)	1.00
**Education**	
None	0.45 (0.36–0.57)***
1–11 years	0.69 (0.56–0.83)***
12+ complete (ref)	1.00
**Employed in the past 12 months**	
Yes	1.51 (1.21–1.90)***
No (ref)	1.00
**Wealth quintile (six city)**	
Poorest	0.94 (0.76–1.16)
Poor	0.88 (0.71–1.10)
Medium	0.96 (0.77–1.19)
Rich	0.93 (0.74–1.17)
Richest (ref)	1.00
**Caste**	
Scheduled caste/tribe	0.63 (0.50–0.79)***
Other backward/extreme backward caste	0.73 (0.61–0.86)***
None (ref)	1.00
**Religion**	
Muslim	0.41 (0.34–0.49)***
non-Muslim (ref)	1.00
**City**	
Agra	0.72 (0.57–0.92)**
Aligarh	0.99 (0.79–1.24)
Allahabad	1.16 (0.91–1.47)
Gorakhpur (ref)	1.00
Moradabad	0.78 (0.63–0.98)*
Varanasi	1.00 (0.79–1.27)
**Residence**	
Slum	0.94 (0.81–1.10)
Non-slum (ref)	1.00
**Parity**	
0–1	0.02 (0.02–0.03)***
2	0.31 (0.24–0.39)***
3	0.43 (0.33–0.57)***
4+ (ref)	1.00

Unweighted n = 11,014; +p ≤ .10; *p ≤ .05; **p ≤ .01; ***p ≤ .001.

Table 4 presents multinomial logistic regression models which explore the patterns of FP use by family sex composition, controlling for demographic factors. In these models, women with no living children and women with zero sons but one or more daughters are significantly less likely to use modern FP than to be a non-user, as compared to women with both sons and daughters but more daughters than sons, controlling for all other factors. Similarly, women with no living children and women with zero sons but one or more daughters were less likely to be a traditional user than a non-user as compared to women with both sons and daughters but more daughters than sons. Women with both sons and daughters but more sons than daughters are significantly more likely to be modern method users than non-users and more likely to be modern users than traditional users as compared to women with both sons and daughters but more daughters than sons. The model presented in Table 4 controls for demographic factors which generally went in expected directions. Notably, less educated women, younger women, and Muslim women were significantly less likely to use modern or traditional contraception than to be a non-user.

**Table 4:** Multinomial logistic regression findings for family planning use among married, non-pregnant fecund women aged 15–49

**Variables**	**Modern user vs. Non-user**		**Traditional user vs. Non-user**		**Modern user vs. Traditional user**	
	**β**	**SE**	**β**	**SE**	**β**	**SE**
**Family sex composition**						
No living children	-2.82	0.15***	-2.78	0.21***	-0.04	0.23
Zero sons, one or more daughters	-0.48	0.09***	-0.38	0.11***	-0.10	0.10
Zero daughters, one or more sons	0.02	0.08	-0.11	0.10	0.13	0.09
Equal number of sons and daughters	0.15	0.08+	-0.00	0.10	0.15	0.08+
Have both sons and daughters, but have more daughters than sons (ref)						
Have both sons and daughters, but have more sons than daughters	0.36	0.07***	0.09	0.09	0.27	0.07***
**Age group**						
15–24	-0.65	0.09***	-0.29	0.11**	-0.36	0.09***
25–29	-0.37	0.08***	-0.25	0.09**	-0.12	0.08
30–34	-0.12	0.07+	-0.08	0.09	-0.04	0.07
35–39	0.04	0.07	0.10	0.09	-0.07	0.07
40+ (ref)						
**Education**						
None	-1.02	0.07***	-0.62	0.08***	-0.40	0.07***
1–11 years	-0.44	0.07***	-0.33	0.08***	-0.11	0.06+
12+ complete (ref)						
**Employed in the past 12 months**						
Yes	0.09	0.07	0.14	0.08+	-0.04	0.07
No (ref)						
**Wealth quintile (six city)**						
Poorest	-0.17	0.07*	-0.08	0.09	-0.09	0.07
Poor	-0.21	0.07**	-0.24	0.08**	0.03	0.07
Medium	-0.08	0.07	-0.14	0.09	0.06	0.07
Rich	-0.20	0.07**	-0.18	0.08*	-0.02	0.07
Richest (ref)						
**Caste**						
Scheduled caste/tribe	-0.10	0.07	0.00	0.09	-0.11	0.08
Other backward/extreme backward caste	-0.09	0.05	-0.04	0.06	-0.05	0.06
None (ref)						
**Religion**						
Muslim	-0.53	0.06***	-0.33	0.07***	-0.21	0.07**
non-Muslim (ref)						
**City**						
Agra	0.00	0.09	-0.26	0.10**	0.27	0.08***
Aligarh	-0.33	0.09***	-0.14	0.09	-0.20	0.09*
Allahabad	0.00	0.09	-0.10	0.11	0.10	0.09
Gorakhpur (ref)						
Moradabad	0.23	0.08**	-0.21	0.10*	0.45	0.08***
Varanasi	-0.04	0.09	-0.74	0.11***	0.70	0.10***
**Residence**						
Slum	-0.12	0.05*	-0.12	0.06+	-0.00	0.05
Non-slum (ref)						
**Parity**						
0–1	-0.98	0.10***	-0.35	0.12**	-0.62	0.11***
2	-0.44	0.08***	0.03	0.10	-0.48	0.09***
3	0.03	0.07	0.06	0.09	-0.03	0.07
4+ (ref)						

Unweighted n = 14,886; +p ≤ .10; *p ≤ .05; **p ≤ .01; ***p ≤ .001.

Table 5 presents the FP method mix by family sex composition among women using a FP method. Overall, female sterilization is the most commonly used method of contraception, followed by condoms and traditional methods. Use of female sterilization is highest among families with both sons and daughters but more sons than daughters and families with both sons and daughters but more daughters than sons, at 56.9% and 48.4%, respectively. Use of female sterilization is lowest among women with no living children and women with zero sons but one or more daughters, at 0.0% and 8.1% respectively; among these two family sex composition categories, the most commonly used modern method was condoms at 64.2% and 46.0%, respectively. The chi square p-value is p ≤ 0.001, indicating that FP method mix differs by family sex composition.

**Table 5:** Percentage of married, non-pregnant fecund women aged 15–49 using a family planning method, by family sex composition

**Variables**	**No living children**	**Zero sons, one or more daughters**	**Zero daughters, one or more sons**	**Equal number of sons and daughters**	**Have both sons and daughters, but have more daughters than sons**	**Have both sons and daughters, but have more sons than daughters**	**Total**
**Method mix** (n = 11,043)* ‡							
Female sterilization	0.0	8.1	27.3	33.2	48.4	56.9	36.1
Male sterilization	0.0	0.5	0.1	0.2	0.2	0.3	0.2
Pill	1.0	5.4	5.0	5.5	5.0	3.8	4.9
IUD	0.0	4.8	5.8	6.2	2.2	1.7	4.2
Injectables	0.0	1.3	0.9	0.7	0.3	0.6	0.7
Condom	64.2	46.0	34.7	29.3	22.3	17.9	29.3
Other modern	0.0	1.9	0.8	0.8	0.7	0.6	0.9
Traditional	34.8	32.1	25.5	24.0	21.0	18.2	23.7
**Total**	100.0	100.0	100.0	100.0	100.0	100.0	100.0

*All percentages and n’s are weighted; ‡F statistic p-value is p ≤ .001.

## Discussion

This study explores son preference using fertility behaviors and family sex composition in urban Uttar Pradesh, India, a state with little research on son preference in urban areas. These findings confirm that family sex composition affects fertility behavior and also reveals that preference for sons persists in urban Uttar Pradesh. Multivariate analyses showed that family sex composition is associated with women’s desire for children; women who have no living children, zero sons but one or more daughters and zero daughters but one or more sons are all significantly less likely to want no more children compared to women that have both sons and daughters but more daughters than sons. Women that have both sons and daughters but more sons than daughters are significantly more likely to want no more children compared to women that have both sons and daughters but more daughters than sons. As the multivariate results show, family sex composition is associated with FP use; women without living children and those with no sons but one or more daughters are less likely to be modern users than nonusers of FP as compared to women that have both sons and daughters but more daughters than sons. Additionally, women with both sons and daughters but a greater number of sons are more likely to be modern method users than nonusers as compared to women with both sons and daughters but a greater number of daughters. This suggests that once women have the number of boys they desire, they rely on modern methods, but also are more likely to use any FP method.

These findings are consistent with other studies in South Asia that show that women with more sons are more likely to want no more children and more likely to be modern FP users [22,29-32]. For instance, in the Demographic Health Survey analysis of three South Asian countries by Jayaraman *et al.* (2009), the study showed that in North India, across all parities, the desire to have another child declined as the number of sons increased and additionally, FP use increased as the number of sons increased [29]. A study using India National Family Health Survey data by Arnold *et al.* (1998) used a simplified measure of family sex composition, comparing women with two sons to women with two daughters among women with only two children, and showed that women with two sons were much less likely to want another child and more likely to use contraception than women with two daughters [22].

These findings also show that women who have more sons are more likely to use sterilization, a long acting, permanent FP method, as compared to women with more daughters. Traditional method use and condom use is highest among women with no living children and women with zero sons but one or more daughters. Therefore, even though the woman is using FP, she is using less effective methods. This may suggest that the woman is willing to take a pregnancy risk of a less effective method if she does not have the number of sons she desires.

Despite the evidence of son preference in South Asia, this study reflects that women may also desire a family sex composition that includes both boys and girls. Families with zero daughters and one or more sons were significantly less likely to want no more children as compared to women with both sons and daughters but more daughters than sons, but the results were not significant for women with an equal number of sons and daughters. This finding is consistent with other studies that suggest girls are also valued [23,31,32,38,39]. A study in rural South Asia showed that couples desired families that were comprised of at least one son and one daughter [32].

The desire for both sons and daughters is further shown by looking at the contraceptive methods used. Sterilized women have ended their reproduction and therefore help show what is the preferred family sex composition. The majority of women that have undergone sterilization have both sons and daughters, with the largest group being women that have both sons and daughters but more sons than daughters, thus suggesting that women did not end their reproduction until they had reached their desired family composition, which included daughters.

These findings are unique in that they investigate son preference in urban Uttar Pradesh, where the effect of lower total fertility and increased modernization on son preference is not well understood. A number of studies investigating son preference in India include rural and urban areas in their sample [22,24,26,40,41], but to the authors’ knowledge, none focus on a large, urban sample of women in Uttar Pradesh as we did here. Das Gupta (2010) posits that modernization and urbanization reduce the influence and strength of patrilineal values, resulting in increased autonomy for urban women [5,7]. Evidence from South Korea has shown that urban women are often able to maintain connections with their own families due to increased independence and autonomy, thus allowing them to continue to play a familial role in their natal home despite being part of their marital home [5]. This continued support to her parents is thought to reduce the incentives for preferring sons over daughters [5]. Conversely, societies that have transitioned to a lower total fertility are also hypothesized to bring about increased preference for sons and sex selective behavior, as women wanting smaller families may desire more control over the sex composition of their families [40]. Additionally, urban areas offer better access to medical technologies [33], and despite Indian government legislation banning prenatal sex determination [8], women in urban areas may use these medical technologies to control their family sex composition. The findings from this study show that women in urban UP have a preference for sons, though the finding that women also want a family composition that includes girls may suggest that preferential attitudes towards sons are attenuated in this population.

This study has a number of limitations worth noting. The first limitation is that family planning use is self-reported. Women may be more likely to over- or under- report use of FP if they are trying to be discrete about FP use or believe that is the desired response. Additionally, cross-sectional data were used for this analysis which allowed for the examination of associations between variables but does not permit an assessment of the direction of causality. Additionally, women’s fertility desires are fluid, and may change over time. Therefore, women may be ambivalent about whether to have another child [42]. Finally, the measure of sex preference is limited to current family sex composition as reported in the baseline survey. An additional limitation is that this study was not able to capture information on sex selective abortion, which means that any of these efforts to control the sex composition of their families are not reflected in this analysis.

One strength of this study is that it uses a large, representative sample of women from six cities in Uttar Pradesh, which allows for in-depth analyses into son preference in urban areas. An additional strength is that the analyses used family sex composition for investigation into son preference, rather than using measures based on hypothetical or ideal number of children that a woman would want. The ideal number of children measure asks women to remember the time when they did not have any children and say what would be her ideal number of children and the sex of these children. This would likely be influenced by her current number of children and the sex of these children, and therefore is not a very accurate measure of son preference. An additional strength of this study is the way that family sex composition is measured. Many of the previous studies explore different categorizations of family sex composition and its relationship with son preference, such as focusing on separate models for each level of parity and sex composition [24,27,29,30], or the ratio of girls to boys [22,24]. The family sex composition variable in this analysis reduces sample selection bias by including women of all parity levels in the models in an effort to present a harmonious model that is relatively easy to interpret.

These findings are important for the design of future family planning programs in urban India. Efforts to increase modern FP use need to be cognizant of the role of son preference and gender preferential attitudes on FP use and fertility desires even in urban settings. Programs aimed at increasing family planning use need to address son preference and include elements that promote the value of girl children. This is critical because with a decline in the desired family size there is the risk of a continued deficit of girls where son preference is common [40].

Programs that seek to reduce son preference also have beneficial effects on the health and social development of girls who are already born. Population policies and programs need to be comprehensive and integrated with other social development efforts of the government, such as those meant to enhance the value of a girl and those that incentivize girls’ education; these types of activities are currently underway in states such as Tamil Nadu, Haryana and Andhra Pradesh and can be implemented more broadly in urban settings [2]. It will take many years to change intrinsic preferences and increase the value of girls; however, linking parents to particular benefits that the government has designed will, in the immediate term, create an uptake of these schemes and potentially create a more gender unbiased environment within families.

At a broader level, there are a number of progressive laws in India that address the patriarchal structures of marriage, dowry, and inheritance which underlie son preference [2]. While not a direct mandate of family planning programs, creating awareness of these laws can unequivocally have a beneficial effect on gender attitudes and son preference in India and lead to increased family planning use so that all women, men and couples attain their desired family size.

## Competing interests

The authors declare that they have no competing interest.

## Authors’ contributions

All authors contributed to the conception and design of the study and participated in the collection of data. LMC performed the statistical analysis and drafted the manuscript. All authors interpreted the data and participated in revision of the manuscript. All authors read and gave final approval for the version submitted for publication.
